# Stem Cell-Like Differentiation Potentials of Endometrial Side Population Cells as Revealed by a Newly Developed *In Vivo* Endometrial Stem Cell Assay

**DOI:** 10.1371/journal.pone.0050749

**Published:** 2012-12-04

**Authors:** Kaoru Miyazaki, Tetsuo Maruyama, Hirotaka Masuda, Akiko Yamasaki, Sayaka Uchida, Hideyuki Oda, Hiroshi Uchida, Yasunori Yoshimura

**Affiliations:** Department of Obstetrics and Gynecology, School of Medicine, Keio University, Tokyo, Japan; University of Cincinnati, United States of America

## Abstract

**Background:**

Endometrial stem/progenitor cells contribute to the cyclical regeneration of human endometrium throughout a woman's reproductive life. Although the candidate cell populations have been extensively studied, no consensus exists regarding which endometrial population represents the stem/progenitor cell fraction in terms of *in vivo* stem cell activity. We have previously reported that human endometrial side population cells (ESP), but not endometrial main population cells (EMP), exhibit stem cell-like properties, including *in vivo* reconstitution of endometrium-like tissues when xenotransplanted into immunodeficient mice. The reconstitution efficiency, however, was low presumably because ESP cells alone could not provide a sufficient microenvironment (niche) to support their stem cell activity. The objective of this study was to establish a novel *in vivo* endometrial stem cell assay employing cell tracking and tissue reconstitution systems and to examine the stem cell properties of ESP through use of this assay.

**Methodology/Principal Findings:**

ESP and EMP cells isolated from whole endometrial cells were infected with lentivirus to express tandem Tomato (TdTom), a red fluorescent protein. They were mixed with unlabeled whole endometrial cells and then transplanted under the kidney capsule of ovariectomized immunodeficient mice. These mice were treated with estradiol and progesterone for eight weeks and nephrectomized. All of the grafts reconstituted endometrium-like tissues under the kidney capsules. Immunofluorescence revealed that TdTom-positive cells were significantly more abundant in the glandular, stromal, and endothelial cells of the reconstituted endometrium in mice transplanted with TdTom-labeled ESP cells than those with TdTom-labeled EMP cells.

**Conclusions/Significance:**

We have established a novel *in vivo* endometrial stem cell assay in which multi-potential differentiation can be identified through cell tracking during *in vivo* endometrial tissue reconstitution. Using this assay, we demonstrated that ESP cells differentiated into multiple endometrial lineages in the niche provided by whole endometrial cells, indicating that ESP cells are genuine endometrial stem/progenitor cells.

## Introduction

Human endometrium lines the uterine cavity and regenerates, differentiates and regresses with each menstrual cycle under hormonal control throughout the course of a woman's reproductive life [1]. These morphological and functional features of human endometrium can be reproduced in an *in vivo* endometrial regeneration model in which severely immunodeficient NOD/SCID/γcnull (NOG) mice are xenotransplanted with dispersed human endometrial cells under the kidney capsule followed by hormonal treatment [2]. It is likely that the cyclical regeneration of human endometrium is achieved through tissue-specific stem cell system(s) in which human endometrium contains a pool of multipotent stem cells capable of cyclically producing progenitor cells that further differentiate into each endometrial cell component [3], [4]. Indeed, several investigators (including our group) have provided evidence suggesting the existence of endometrial stem/progenitor cell cells and their possible roles in humans [5], [6]. The candidate endometrial stem/progenitor cells include clonogenic endometrial cells [7], endometrial side population (SP) cells that possess a Hoechst 33342 low-fluorescence profile [8]–[11], CD146^+^CD140b^+^ stromal cells [12], CD29^+^CD73^+^CD90^+^ stromal cells [13], and W5C5^+^ stromal cells [14]. There exists, however, no consensus regarding which endometrial population represents the stem/progenitor cell fraction [6], [15].

Tissue-specific, candidate stem cells have been identified in many types of tissues based on the SP phenotype [16]. This characteristic is due to the unique ability of the primitive cells to pump out the DNA-binding dye Hoechst 33342 via the ATP-binding cassette transporter G2 (ABCG2) [16]. Primitive hematopoietic precursors from bone marrow were the first SP cells identified with this technique [17]. We previously demonstrated that SP cells, but not main population (MP) cells, both isolated from the human endometrium, regenerate human endometrium-like tissues *in vivo* when xenotransplanted under the kidney capsule of NOG mice [10]. However, a low efficiency of reconstitution was observed, possibly because endometrial SP (ESP) cells may require a specific “niche” provided by other endometrial cell components to reconstitute the entire endometrium *in vivo* as well as in *in vitro* culture. Indeed, successful proliferation of ESP cells in the presence of endometrial MP (EMP) cells in conventional media [10] suggests that EMP may provide a “niche” appropriate for activation of ESP.

In the present study, we modified and improved our previous *in vivo* endometrial regeneration model [2] to characterize the stem cell properties of human ESP cells. Using our newly developed *in vivo* endometrial stem cell assay, we have demonstrated that ESP cells are significantly more capable of differentiating into multiple endometrial lineages than are EMP cells in assistance with the niche provided by whole endometrial cells.

## Results

### Isolation of ESP and EMP Cells from Human Endometrium

**Figure 1:**
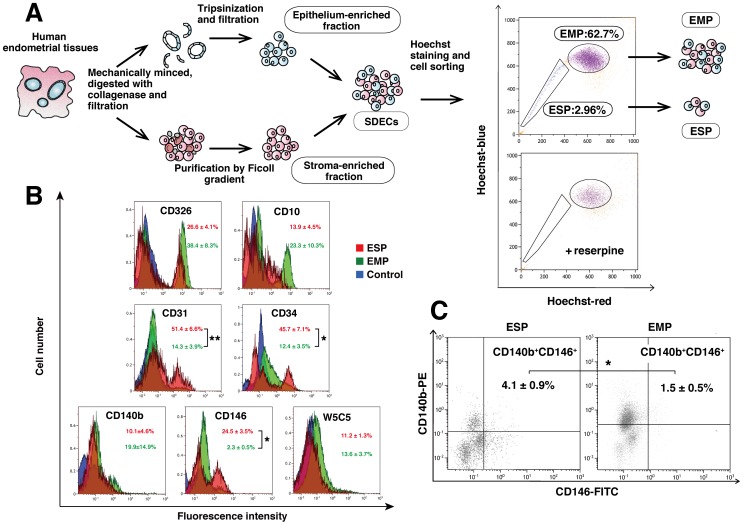
Isolation and cell surface marker characterization of ESP and EMP. (**A**) Summary of procedures for the preparation of epithelium-enriched and stroma-enriched fractions from cycling human endometrium and isolation of ESP and EMP cells from the mixture of both fractions (SDECs). The two right-hand panels illustrate the representative images of flow cytometric distribution of ESP and EMP in SDECs stained with Hoechst 33342 in the absence (upper) and presence of 50 µM reserpine (lower). (**B**) Flow cytometric analysis of the expression patterns of surface markers for epithelial cells (CD326), stromal cells (CD10), endothelial cells (CD31 and CD34), and endometrial mesenchymal stem-like cells (CD140b, CD146 and W5C5) in ESP (red), EMP (green) and unstained controls (blue). Histograms are representatives of six independent recipients. Values are expressed as means ± SEM. * *P*<0.005. ** *P*<0.01. (**C**) The percentage of CD140B^+^CD146^+^ cells in ESP (left) and EMP cells (right). Dotplots are representatives of six independent recipients. Values are expressed as means ± SEM. * *P*<0.05.

As shown in Figure 1A, we first dissociated human endometria mechanically and enzymatically and purified epithelia-enriched and stroma-enriched fractions as previously described [1]. These two fractions were mixed, and the mixture (designated as singly dispersed endometrial cells (SDECs)) was then stained with Hoechst dye followed by flow cytometric analysis and cell sorting for isolation of ESP and EMP cells (Figure 1A, right upper panel). The ESP fraction disappeared upon treatment with 50 µM reserpine, an ABCG2 blocker (Figure 1A, right lower panel). The percentage of ESP cells among SDECs was 3.13±0.61% (mean±SEM, n = 8).

### Surface Marker Characterization of ESP and EMP Cells

We next characterized the expression patterns of several cell surface markers in ESP cells in comparison with EMP cells. As shown in Figure 1B, there were no significant differences in the percentages of cells positive for CD326 (epithelial) [18] and CD10 (stromal) [19] expression between ESP and EMP cells (CD326, 26.6±4.1% vs. 38.4±8.3%; CD10, 13.9±4.5% vs. 23.3±10.3%; n = 6, each group). The expression levels of CD31 (endothelial), CD34 (endothelial, hematopoietic stem/progenitor), and CD146 (endothelial, mesenchymal stem/progenitor) [12] were significantly higher in ESP than in EMP cells (CD31, 51.4±6.6% vs. 14.3±3.9%, *P*<0.01; CD34, 45.7±7.1% vs. 12.4±3.5%, *P*<0.005; and, CD146, 24.5% ±3.5 vs. 2.3±0.5%, *P*<0.005; n = 6, each group) (Figure 1B). There was no difference in the percentages of W5C5-positive cells, purportedly putative human endometrial mesenchymal stem-like cells [14], between ESP and EMP fractions (11.2±1.3% vs. 13.6±3.7%; n = 6, each group) (Figure 1B). CD140b^+^CD146^+^ cells, reportedly putative human endometrial mesenchymal stem-like cells [12], were significantly more abundant in the ESP fraction than in EMP cells (4.1±0.9% vs. 1.5±0.5%, *P*<0.05; n = 6, each group) (Figure 1C). Thus, flow cytometric analysis demonstrated that ESP and EMP fractions contained similar proportions of epithelial and stromal marker-positive cells; however, endothelial cells and mesenchymal stem-like cells were significantly more abundant in ESP than in EMP cells.

### *In vivo* Stem Cells Assay Using ESP and EMP Fractions

ESP cells alone can give rise to endometrium-like tissues with delineated glandular structures *in vivo* when xenotransplanted under the kidney capsule of NOG mice [10]. However, the reconstitution efficiency has been very low (≤10%). We suggest that this is because ESP cells require a “niche” provided by other endometrial cell components to reconstitute the entire endometrium *in vivo* and also to proliferate and differentiate into various endometrial lineages *in vitro*. Indeed, co-culture with EMP cells improves the poor proliferative potential of ESP cells [10] suggesting that the EMP fraction may provide a “niche” essential for elicitation of ESP stem cell activity.

**Figure 2:**
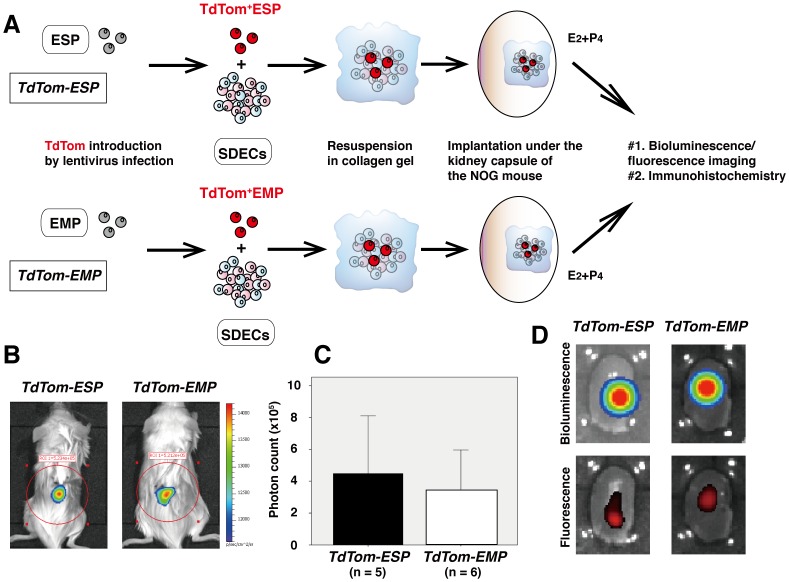
*In vivo* human endometrial stem cell assay using ESP and EMP. (**A**) Summary of procedures for *in vivo* endometrial stem cell assay. E_2_+P_4_, treatment with estradiol in combination with progesterone. (**B**) Representative BLI of a ventrally positioned NOG mouse one week after xenotransplantation using suspensions harboring the endometrial constructs expressing RedFluc derived from *TdTom-ESP* (left panel) or *TdTom-EMP* (right panel). (**C**) Quantitative assessment of BLI signals derived from the reconstituted endometrial tissues containing *TdTom-ESP* (n = 5) or *TdTom-EMP* (n = 6). Each bar indicates the mean+SEM. (**D**) Representative BLI (upper two panels) and fluorescence images (lower two panels) of kidneys excised from NOG mice transplanted with *TdTom-ESP* (left two panels) or *TdTom-EMP* (right two panels).

These ideas and data prompted us to develop an *in vivo* endometrial stem cell assay in which multilineage differentiation of endometrial stem cells could be identified through cell tracking during endometrial tissue reconstitution. Figure 2A illustrates the procedures for the *in vivo* stem cell assay. Stem or non-stem cells – ESP or EMP cells in this study – were infected with lentivirus immediately after cell sorting without cell culture to express bioluminescent/fluorescent marker(s). Specifically, we used red-emitting firefly luciferase (RedFluc) and tandem Tomato (TdTom) that can be detected by bioluminescence and fluorescence imaging (BLI and FLI, respectively). The infected cells were mixed with SDECs, resuspended in collagen gel, and then implanted under the kidney capsule of ovariectomized NOG mice. These mice were treated with estrogen and progesterone for about eight weeks, and then subjected to BLI followed by nephrectomy. The graft-bearing kidneys were also subjected to BLI and FLI, histological and immunohistochemical analyses. The NOG mice (and their kidneys) transplanted with TdTom-expressing ESP cells and TdTom-expressing EMP cells were designated *TdTom-ESP* and *TdTom-EMP*, respectively.

We performed BLI of the ventrally positioned *TdTom-ESP* and *TdTom-EMP* mice one week after xenotransplantation and detected bioluminescent signals in locations corresponding to the right kidney (Figure 2B). Quantification of the measured light revealed that the signal intensities reflecting the number of RedFluc-expressing cells were not significantly different between the *ESP-TdTom* mice (4.28±2.33×10^5^ photon count, n = 5) and the *EMP-TdTom* mice (3.43±1.26×10^5^ photon count, n = 6) (Figure 2C). BLI and FLI analysis of NOG mouse kidneys excised eight weeks after xenotransplantation clearly showed that intense focal bioluminescent and fluorescent spots were confined to the transplanted sites (Figure 2D) indicating the existence of TdTom-labeled ESP and EMP cells or their descendants in these kidneys.

### Histological and Immunohistochemical Analysis of *in vivo* Reconstituted Tissues

**Figure 3:**
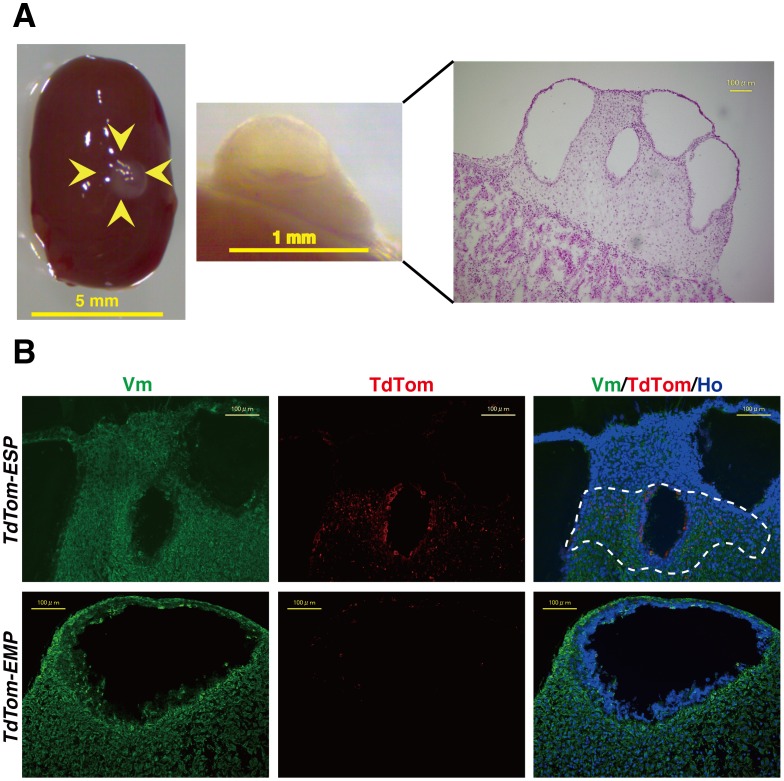
Macroscopic and microscopic findings of human endometrium-like tissues reconstituted in the *in vivo* stem cell assay. (**A**) Representative macroscopic images (left two panels) of the transplanted site (arrowheads) of NOG mice eight weeks after xenotransplantation of *TdTomato-ESP*. H&E staining was performed on the transplanted lesion (right; scale bar, 100 µm). (**B**) Representative immunofluorescence staining of the endometrial constructs derived from *TdTomato-ESP* (upper panels) and *TdTomato-EMP* (lower panels) using antibodies against vimentin (Vm), and TdTom followed by Hoechst staining. Note that localization of TdTom-expressing cells was focal as surrounded by a dotted line in the endometrial constructs derived from *TdTomato-ESP*, whereas much less TdTom-expressing cells were sporadically distributed in *TdTomato-EMP*-derived constructs. Scale bars, 100 µm.

Detectable bioluminescent and fluorescent signals indicate not only the presence of the labeled cells but also the existence of reconstructed tissue containing those cells in the kidney. Indeed, an apparent cystic mass was macroscopically observed at the site of the transplant used for the *in vivo* stem cell assay (Figure 3A, left and middle panels). H&E staining revealed that the cystic mass possessed well-delineated glands and stroma like endometrium (Figure 3A, right panel). The mass was positive for human vimentin (Vm), as demonstrated by immunofluorescence studies using anti-human vimentin (Vm) antibody that recognizes only human Vm, a representative marker for endometrial stromal cells.

In this stem cell assay, ESP or EMP cells (1×10^4^ each) were infected with lentivirus without cell culture to introduce TdTom and then mixed with propidium iodide (PI)-negative SEDCs (4.9×10^5^ cells each). In contrast to ESP alone as previously reported [10], the efficiency of endometrial tissue reconstitution was 100% (n = 11) when the cell mixture was used for transplantation. Since the majority (approximately 98%) of the initial mixture was SDECs, it is quite reasonable that the high reconstitution efficiency was the same as that obtained in the previous study in which only SEDCs were used for *in vivo* reconstitution assay. In other words, lentivirally TdTom-marked ESP and EMP cells did not affect the reconstitution potentials of SEDCs. Rather, TdTom-positive ESP were focally present in the reconstituted tissue as shown by a dotted line in Figure 3B (middle and right panels), collectively suggesting that ESP cells focally contributed to the endometrial tissue reconstitution driven by non-labeled SDECs.

We next investigated which fraction, ESP or EMP, was more involved in the endometrial regeneration and which endometrial lineage they differentiated into. Thus, to determine how ESP and EMP contribute to the genesis of each endometrial linage, we performed fluorescent co-staining of TdTom in combination with each of the differentiation markers including Vm (stroma), cytokeratin (Ck, epithelium), α-smooth muscle actin (α-SMA, smooth muscle), and CD31 (endothelium) to identify the behavior of TdTom-labeled cells.

**Figure 4:**
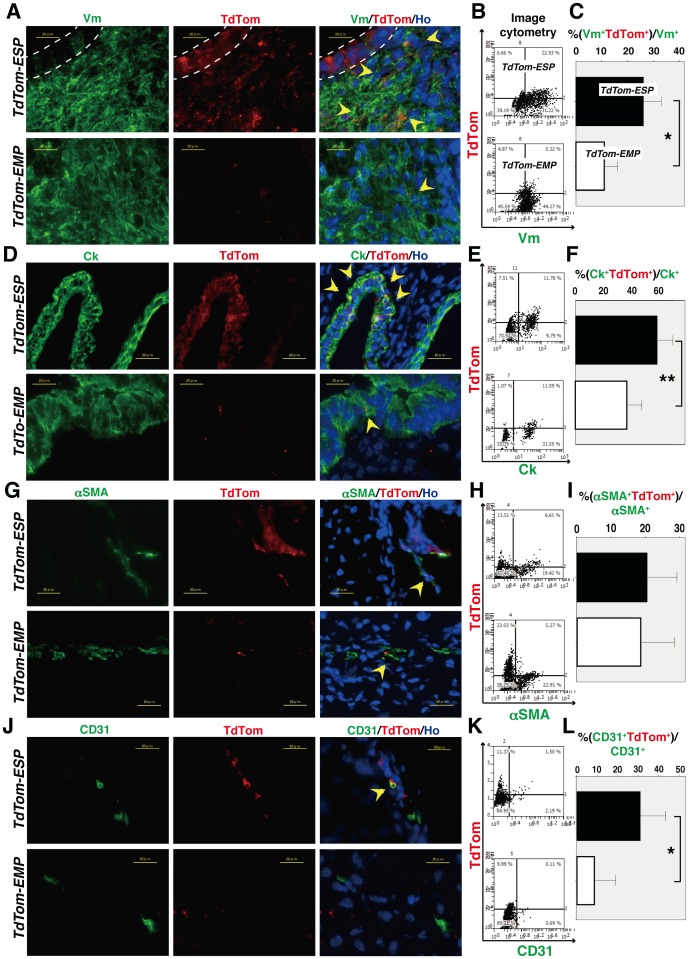
Co-expression of each endometrial lineage marker and TdTom in *TdTom-ESP* and *TdTom-EMP* kidneys. Representative immunofluorescence images of the *TdTom-ESP*- and *TdTom-EMP*-derived reconstituted endometrial tissues immunostained with anti-TdTom antibody together with antibodies against vimentin (Vm) (**A**), cytokeratin (Ck) (**D**), α-smooth muscle actin (α-SMA) (**G**), and CD31 (**J**). Nuclei were stained with Hoechst dye. Yellow arrowheads indicate cells co-expressing TdTom and the corresponding endometrial lineage marker (**A**, **D**, **G**, and **J**). Typical scattergrams from microscopic analyses of co-localization of TdTom and an endometrial lineage marker such as Vm (**B**), Ck (**E**), α-SMA (**H**), or CD31 (**K**) in reconstituted tissues derived from *TdTomato-ESP* or *TdTomato-EMP*. The horizontal and vertical axes of the scattergrams indicate the intensities of Alexa 488 (green, lineage marker) and Alexa 555 (red, TdTom). We set the cutoff point referring intensity of mouse kidney parenchyma in each section as controls. Black and white bar graphs – *TdTomato-ESP* and *TdTomato-EMP*, respectively – indicate the mean+SEM of the percentage of cells doubly positive for TdTom and Vm (**C**), Ck (**F**), α-SMA (**I**), or CD31 (**L**) among whole TdTom^+^ cells, as determined by image analysis using TissueQuest software (n = 36 visual fields per group). Bars, 20 µm. * *P*<0.0005, ** *P*<0.01. Note that TdTom-positive epithelial cells, which are surrounded by dotted lines, localize adjacent to TdTom positive stromal cells in (A).

Immunofluorescence clearly showed that TdTom^+^Vm^+^, TdTom^+^Ck^+^ and TdTom^+^CD31^+^ cells were more abundant in the reconstituted tissues derived from TdTom^+^ ESP cells than in those from TdTom^+^ EMP cells (Figure 4, A, D, and J). There was no difference in the number of TdTom^+^α-SMA^+^cells between the TdTom^+^ ESP fraction and the TdTom^+^ EMP fraction (Figure 4G). Endometrial epithelial cells exhibited evenly distributed TdTom signals, whereas the other types of endometrial cells, particularly stromal cells, displayed tiny dot signals (Figure 4 and Figure S1).

The percentage of cells doubly positive for an endometrial lineage marker and TdTom among lineage marker^+^ cells reflects the potential of TdTom^+^ ESP and EMP fractions for differentiation into the corresponding endometrial lineage. To quantify the percentage of endometrial lineage-positive TdTom^+^ cells in the immunofluorescent slides, we performed an image analysis of 36 visual fields (three visual fields per section, three sections per sample, four samples) from each differentiation marker by TissueQuest software (Tissue Gnostic) and show the representative scattergrams for each endometrial linage (Figure 4, B, E, H, and K). In agreement with results of the qualitative analysis of immunofluorescence signals, the quantitative “TissueQuest“ analysis revealed that the percentages of TdTom^+^Vm^+^, TdTom^+^Ck^+^ and TdTom^+^CD31^+^ cells among each lineage marker^+^ cells were significantly higher in the reconstituted tissues originated from TdTom^+^ ESP cells than in those from TdTom^+^ EMP cells. Specifically, the data showed the following: TdTom^+^Vm^+^, 26.2±3.4% vs. 11.0±2.6%, *P*<0.0005; TdTom^+^Ck^+^, 58.7±5.6% vs. 36.9±5.3%, *P*<0.01; TdTom^+^CD31^+^, 31.9±6.0% vs. 6.8±4.3%, P<0.0005, Figure 4, C, F, and L. In contrast, there was no difference in the percentages of TdTom^+^α-SMA^+^ cells between TdTom^+^ ESP and TdTom^+^ EMP fractions (27.8±5.5% vs. 19.3±4.5%; Figure 4H). Thus, these results collectively suggest that ESP cells have *in vivo* multi-lineage differentiation potentials characteristic of somatic stem cells and that they are capable of differentiating into endometrial stromal, epithelial, smooth muscle, and endothelial cells.

**Figure 5:**
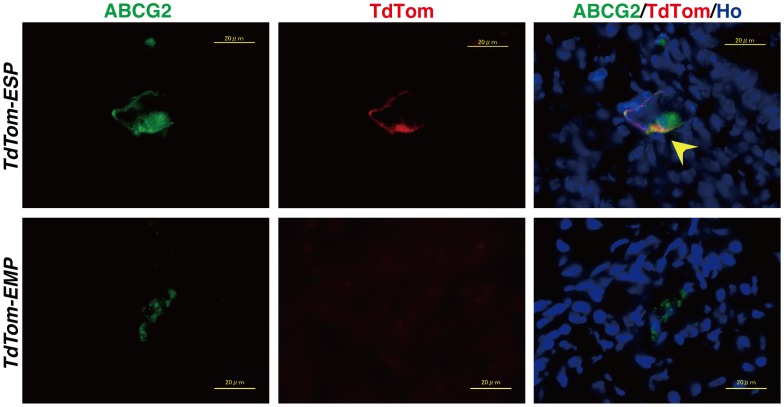
Co-expression of ABCG2 and TdTom in *TdTom-ESP*, but not *TdTom-EMP*. Representative immunofluorescent images of the *TdTom-ESP*- and *TdTom-EMP*-derived reconstituted endometrial tissues immunostained with anti-TdTom antibody together with an antibody against ABCG2. A yellow arrowhead indicates a cell co-expressing TdTom and ABCG2. Bars, 20 µm.

ESP cells, but not EMP cells, are known to preferentially and exclusively express ABCG2, a stem cell marker [19]. To trace the stem cell-like behavior of transplanted TdTom^+^ cells, we investigated co-expression of ABCG2 and TdTom in the reconstituted tissue. Immunofluorescence studies using antibodies against ABCG2 and TdTom revealed that very few but significant numbers of double positive cells could be detected. Importantly, they were observed only in *TdTom-ESP* mice but never in *TdTom-EMP* mice (Figure 5). The intensity of the ABCG2 fluorescence was not strong enough to be subjected to quantitative “TissueQuest” analysis. Taken together these results suggest that the majority of ESP cells may give rise to several endometrial lineages whereas a remnant minor fraction of ESP cells may remain undifferentiated and possibly retain a stem cell phenotype even eight weeks after transplantation.

## Discussion

Many groups (including ours) have identified, isolated, and characterized putative endometrial stem/progenitor cells and their relevant cells through a variety of methods [6], [15]. It seems, however, that each method produces cells that are distinct from the cells isolated by another method [6], [15]. There are several *in vitro* assays to identify and characterize stem cell activity, including clonogenic assays and multi-lineage differentiation assays. In particular, clonal activity is routinely used for the identification of putative somatic stem cells in many adult tissues including the endometrium [20]–[22]. If a particular cell does not have significant clonogenic activity, it may not be considered a stem/progenitor cells. It remains possible, however, that *in vitro* culture lacks the proper conditions for the maintenance of viability, growth, and differentiation. *In vitro* multi-lineage differentiation assays may have similar drawbacks. Thus, an *in vivo* stem cell assay is absolutely needed to identify bona fide stem cells. Therefore, *in vivo* repopulation assays are considered to be stringent approaches to the identification and characterization of hematopoietic stem cells [23]. Indeed, it has been reported that most hematopoietic cells capable of long-term repopulation *in vivo* may not be detected as clonogenic cells in standard culture systems [24], [25].

We previously demonstrated that human ESP cells are capable of regenerating human endometrium-like tissues *in vivo* when xenotransplanted under the kidney capsule of NOG mice [10]. Although the previous study showed that ESP clonal efficiency was low compared to EMP cells [10], the *in vivo* regeneration and multi-lineage differentiation potentials of ESP cells strongly suggest that ESP cells are likely endometrial stem/progenitor cells [10], [26]. The regeneration of well-structured endometrium, however, was inefficient likely because the niche provided by other endometrial cells was lacking. This drawback makes it difficult to compare the differentiation capacity of ESP and EMP cells [10]. We hypothesized that ESP might behave as progenitor/stem cells when surrounded an appropriate microenvironmental niche. Therefore, we developed a novel *in vivo* stem cell assay in which a mixture of ESP cells and whole endometrial cells function together as a transplanted “niche” [2]. For this purpose, EMP cells as the control or ESP cells must be distinguished from the “niche” cells and, therefore, they must be labeled with bioluminescent and fluorescent proteins to track their contribution to each endometrial lineage during endometrial tissue reconstitution. In this assay, we have mixed dispersed cells with a collagen gel and placed the suspension under the kidney capsule using a soft flexible thin tube. This new cell transplantation technique was much easier and much less invasive than our previous procedure in which a simple liquid suspension of dispersed cells was injected through the mouse kidney parenchyma using a micropipette [2].

Using this assay, we have successfully shown a high reconstitution rate, approximately 100%, and demonstrated that ESP cells could differentiate into several endometrial lineages, including epithelial, stromal, and endothelial cells. These results suggest that the ESP fraction may contain a higher proportion of stem/progenitor cells capable of differentiating into endometrial components than does the EMP fraction. Indeed, flow cytometric analysis showed that the proportion of CD140b^+^CD146^+^ cells, reportedly endometrial mesenchymal stem-like cells [12], was significantly higher in ESP cells than in EMP cells. However, it is conceivable that the ESP fraction contains several types of partially differentiated endometrial lineages at higher frequencies than the EMP fraction and that those differentiated cells, in turn, may survive and expand during tissue reconstitution. In this context, one can argue that the magnitude of the contribution may reflect the proportion of pre-existing differentiated cells, but not that of stem/progenitor cells. To address this criticism, we compared the expression of several differentiation markers in ESP and EMP fraction. We found that there were no significant differences at least in CD326^+^ (epithelial) and CD10^+^ (stromal) cells (Figure 1B), suggesting that the higher contribution of ESP cells to epithelium and stroma in recapitulated tissues may be due to an abundance of stem/progenitor cells rather than that of well-differentiated stromal and epithelial cells.

In contrast to epithelial and stromal cells, CD31^+^ (endothelial) and CD34^+^ (endothelial, stem) cells were more abundant in the ESP fraction than the EMP fraction (Figure 1B), which agrees with our previous results [10]. In this context, the abundance of differentiated endothelial cells in ESP cells may result in a higher contribution of ESP to endothelial cells than EMP. CD31^+^ and CD34^+^ cells, however, are not necessarily well-differentiated endothelial cells. Indeed, endothelial progenitor cells are also positive for CD31 and CD34 [27]. Furthermore, we have previously shown that ESP cells exhibit an endothelial progenitor cell-like phenotype [10]. Thus, it is more likely that the stem cell activity of ESP may also contribute to the genesis of endothelium during tissue reconstitution.

ESP cells represent the most likely candidates for endometrial stem/progenitor cells; however, there are two possibilities with regard to the composition of the ESP fraction. First, it could contain “master” uterine stem cell(s) capable of giving rise to almost every type of endometrial lineage. Secondly, there may be progenitors for every type of endometrial lineage present in the ESP fraction and they could generate each type of fully differentiated cell component. The latter possibility is more likely based on the present results that the ESP fraction contains several types of cells positive for epithelial, stromal, and endothelial markers. Our new *in vivo* stem cell assay will allow us to further identify which fraction of the ESPs is responsible for the genesis of each endometrial lineage. Based on the present results showing high stem cell activity of the ESP fraction compared to that of the EMP fraction, we will compare *in vivo* stem cell activity among CD326^+^SP, CD326^−^SP and whole SP. The number of CD326^+^SP is so small that they alone may not be able to give rise to identifiable tissues. However, with assistance from whole endometrial cells as “niche” cells, we expect that CD326^+^SP may be able to contribute to the formation of one or more endometrial lineages.

As shown in Figure 4E, some glands mainly consist of TdTom^+^ cells but other glands consist of both TdTom^+^ and TdTom^−^ cells in *TdTom-ESP* mice. It has been reported that each gland of human eutopic endometrium is monoclonal and therefore may have originated from a single or multiple stem/progenitor cells with uniform clonality at the bottom of each endometrial gland [28]. However, this interpretation seems inconsistent with our present data. Since the disorganized and unpolarized mixtures of non-labeled whole endometrial cells and TdTom-labeled ESP cells are the starting material for endometrial tissue reconstitution in this assay, it is possible that both non-labeled ESP cells present in whole endometrial cell populations and TdTom-labeled ESP may together contribute to the genesis of glands in a disorganized manner therefore resulting in “hybridized” or “mosaic” gland formation.

Endometrial epithelial cells exhibited evenly distributed TdTom signals, whereas the other types of endometrial cells, particularly stromal cells, displayed tiny dot signals (Figure 4 and Figure S1). The difference in the pattern of TdTom signals was not due to an artifact of immunostaining and imaging procedures, because the difference was still observed in the same section (Figure S1). It is possible that the pattern of intracellular distribution of TdTom may depend on the cell type. Indeed, the localization of TdTom varies according to the cell type [29], and DsRed2, another type of RFP, shows dotted pattern in cells derived from rhabdomyosarcoma [30].

In summary, we have established a novel *in vivo* endometrial stem cell assay that enabled us to track the differentiation of the ESP fraction into each endometrial lineage. We observed a high efficiency of endometrial tissue reconstitution. The results of this study further consolidate characterization of the ESP fraction as previously reported [8], [9], [10], [26]. Many groups (including ours) have identified putative endometrial stem/progenitor cells and their relevant cells through a variety of methods [6], [15]. Nevertheless, it remains to be determined how many types of stem/progenitor cells are present in the human endometrium, how they differ in phenotype and function, and the nature of the hierarchical relationships that extend across the various types of precursors. To gain consensus regarding which endometrial population represents *bona fide* stem/progenitor cells in terms of *in vivo* stem cell activity, this newly developed *in vivo* endometrial stem cell assay will be useful for identification and/or validation of human endometrial stem/progenitor cells reported thus far and in the future.

## Materials and Methods

### Ethics Statement

This study was approved by the Keio University Ethics Committee, and informed written consent was obtained from each patient prior to tissue collection. Procedures performed on animals were also approved by the Keio University Ethics Committee and conducted in accord with the Guide for the Care and Use of Laboratory Animals of the Keio University School of Medicine.

### Tissue Collection

Secretory phase endometrial specimens without any abnormalities or malignancies were collected from consenting women (aged 38–49 years) with normal menstrual cycles undergoing total abdominal hysterectomy for benign gynecological diseases. We used two endometrial specimens for grafting and six for flow cytometric analysis. The use of these human specimens was approved by the Keio University Ethics Committee.

### Isolation and Flow Cytometric Analysis of ESP and EMP Cells

Endometrial specimens were separated and dissociated into endometrial stromal and glandular epithelial single cell fractions as described previously [2]. The mixture of two fractions (SDECs) was washed in calcium- and magnesium-free Hanks' Balanced Salt Solutions (HBSS) (Sigma-Aldrich) supplemented with 2% FBS, 10 mM Hepes Buffer Solution (Sigma-Aldrich) (HBSS+) and suspended at 2×10^6^ cells/mL in HBSS+ and stained with 5.0 µg/mL Hoechst 33342 (Sigma-Aldrich) for 90 min at 37°C, as described previously [17], [31]. For flow cytometry, fluorescein isothiocyanate (FITC)-conjugated or phycoerythrin (PE)-conjugated antibodies and propidium iodide (PI) (Sigma-Aldrich) were simultaneously added to Hoechst-stained cells suspended in HBSS+. Cells were incubated on ice for 30 min, pelleted, and washed with HBSS+. The antibodies are listed in Table S1. Flow cytometric analysis and cell sorting were performed as described in Methods S1. After collecting 1×10^5^ events, the SP population was defined as previously reported [17]. Samples were analyzed using Kaluza software (Beckman Coulter).

### Lentiviral Vector

The self-inactivating HIV-1-based lentiviral vector, UBC-RedFluc-T2A-tdtomato, was purchased from Targeting Systems (El Cajon, CA, USA). The vector expresses red-emitting firefly luciferase (RedFluc) under control of the UBC (ubiquitin) promoter and co-expresses tandem Tomato (TdTom), an improved version of red fluorescent protein (RFP), using a T2A.

### Lentiviral Infection of Transduced ESP and EMP Cells and Preparation of Endometrial Grafts

ESP and EMP cells were infected with lentivirus by centrifugation without cell culture as described in Methods S1. Infected ESP or EMP cells (1×10^4^ cells each) were mixed with 4.9×10^5^ PI-negative SDECs and resuspended in rat-tail collagen (BD Biosciences) neutralized according to the manufacturer's instructions, and the mixtures were dispensed in 15 µL aliquots and incubated for 30 min at 37°C as described previously [32]. They were designated *TdTomato-ESP* and *TdTomato-EMP*, respectively. The collagen suspensions were overlaid with DMEM+ and incubated overnight. The following day, *TdTomato-ESP* or *TdTomato-EMP* was implanted under the kidney capsule of oophorectomized NOD/SCID/γcnull (NOG) mice. We used two human specimens and the procedure was performed in duplicate or triplicate for each specimen. Thus, a total of five *TdTomato-ESP*s and six *TdTomato-EMP*s were transplanted.

### Xenotransplantation and Hormonal Treatment

NOG mice [33] were used for xenotransplantation experiments. Either *TdTomato-ESP* or *TdTomato-EMP* was transplanted under the kidney capsule (for details, see Methods S1). At transplantation, both recipients’ ovaries were removed and the recipient was implanted subcutaneously with two E_2_ pellets (1.5 mg of E_2_ per pellet; Innovative Research of America, Sarasota, FL, USA). A P_4_ pellet (15 mg of P_4_ per pellet; Innovative Research of America) was subcutaneously implanted six weeks after the transplantation. These xenotransplanted mice were nephrectomized according to the experimental protocol (for details, see Methods S1).

### Histology and Immunohistochemistry

H&E-staining and immunofluorescence analyses were performed on cryosections derived from kidneys transplanted with TdTomato-ESP or TdTomato-EMP that were air-dried, washed, and fixed. After permeabilization and blocking, tissue sections were incubated with the pre-titrated primary antibodies listed in Table S2. For indirect fluorescence staining, the first antibodies were visualized by incubation with secondary antibodies conjugated with Alexa Fluor 488 (green) (Life Technologies, Carlsbad, CA, USA) and Alexa Fluor 555 (red) (Cell Signaling Technology, Danvers, MA, USA). Images were collected as described in Methods S1. For image cytometry, images were analyzed using the TissueQuest software (TissueGnostics, Vienna, Austria) as described in Methods S1.

### Bioluminescence Imaging (BLI) and Fluorescence Imaging (FLI)

We used a Xenogen-IVIS 100 cooled CCD optical macroscopic imaging system (SC BioScience Corporation, Tokyo, Japan) for BLI and FLI. For *in vivo* BLI, OVX-NOG mice xenotransplanted with lentivirally engineered SDECs were anesthetized with 3% isoflurane and given a retro-orbital injection of D-luciferin (SC BioScience Corporation) (150 mg/kg body weight). All images were analyzed as described in Methods S1. To quantify the measured light, regions of interest (ROI) were defined over the transplanted area and all values were examined from an equal ROI. NOG mouse kidneys excised eight weeks after xenotransplantation were placed on culture dishes and subjected to FLI followed by BLI in the presence of 150 µg/ml D-luciferin.

### Statistics

Statistical analysis was carried out using IBM-compatible SPSS for Windows version 19.0.0 (SPSS Inc., Chicago, IL, USA). Results are expressed as means ± SEM. Distribution of each sample was assessed with the Shapiro-Wilk test. When the distributions of both samples were normal their dispersions were assessed with the Levene test. We compared homoscedastic samples using a two-sample t-test or for heteroscedastic samples, we used the Welch test. When at least either of the samples was not normally-distributed, they were compared using the Mann-Whitney test. P values less than 0.05 were considered statistically significant.

## Supporting Information

Figure S1 **Representative immunofluorescent images of the** ***TdTom-ESP*** **-derived reconstituted endometrial tissues immunostained with anti-TdTom antibody together with an antibody against Ck. Yellow arrowheads indicate epithelial cells and yellow arrows indicate stromal cells. Note that there are TdTom positive stromal cells around epithelial cells and the TdTom signal is evenly observed in epithelial cells whereas dotted signal is seen in stromal cells in the same and condition condition. Bars, 20 µm.**
(TIF)

Table S1 **List of antibodies used for flow cytometric analyses.**
(XLSX)

Table S2 **List of antibodies used for immunofluorescence staining.**
(XLSX)

Methods S1 **Supplemental methods.**
(DOC)
