# Supplementary figures and images for: Stem Cell-Like Differentiation Potentials of Endometrial Side Population Cells as Revealed by a Newly Developed In Vivo Endometrial Stem Cell Assay
Source: PLoS One. 2012 Dec 4;7(12):e50749. doi: 10.1371/journal.pone.0050749 (PMC3514174; doi:10.1371/journal.pone.0050749)

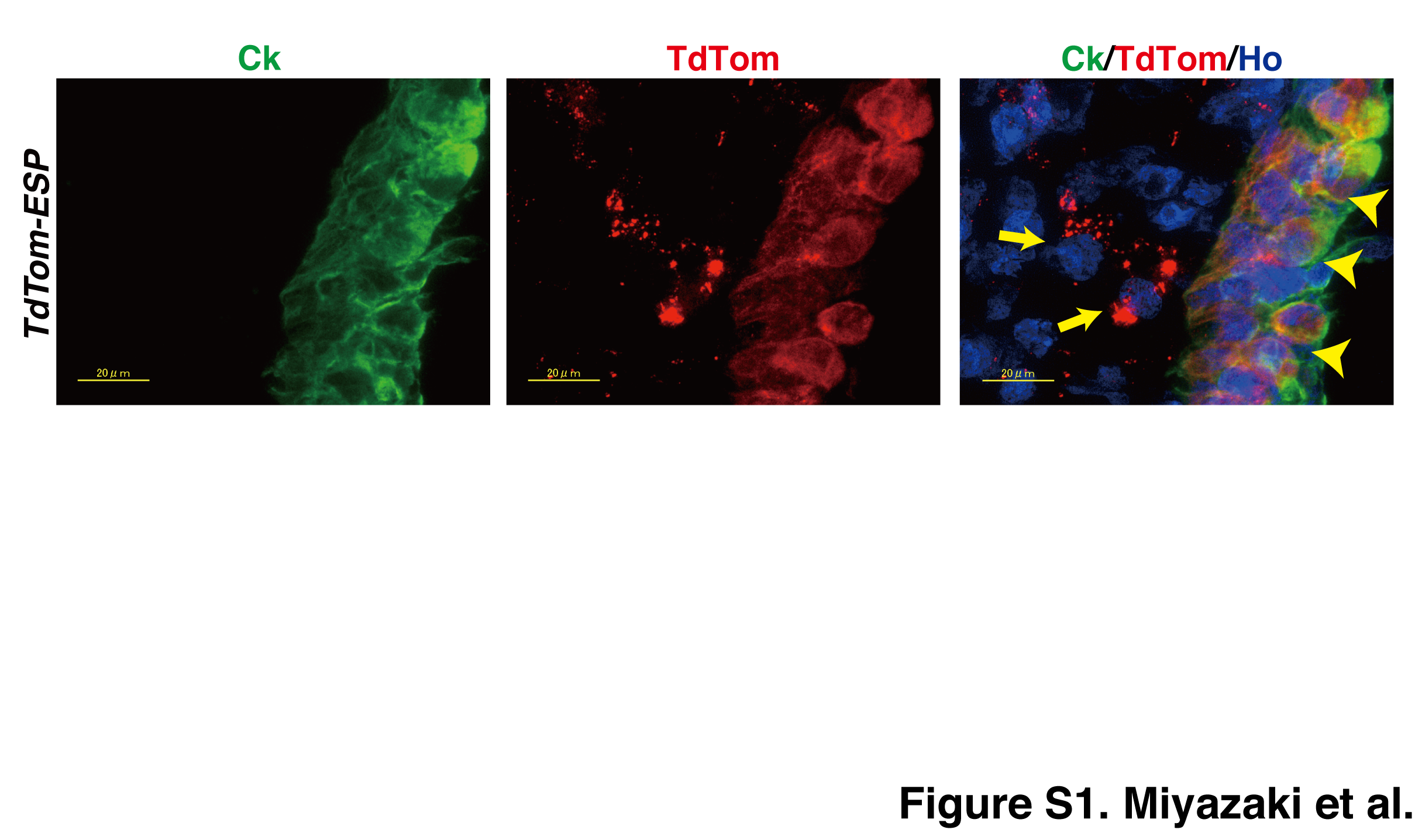

Supplement: Figure S1 — Representative immunofluorescent images of the TdTom-ESP -derived reconstituted endometrial tissues immunostained with anti-TdTom antibody together with an antibody against Ck. Yellow arrowheads indicate epithelial cells and yellow arrows indicate stromal cells. Note that there are TdTom positive stromal cells around epithelial cells and the TdTom signal is evenly observed in epithelial cells whereas dotted signal is seen in stromal cells in the same and condition condition. Bars, 20 µm. (TIF) [file pone.0050749.s001.tif]
