# Supplementary material for: Stem Cell-Like Differentiation Potentials of Endometrial Side Population Cells as Revealed by a Newly Developed In Vivo Endometrial Stem Cell Assay
Source: PLoS One. 2012 Dec 4;7(12):e50749. doi: 10.1371/journal.pone.0050749 (PMC3514174; doi:10.1371/journal.pone.0050749)
Supplement: Methods S1 — Supplemental methods. (DOC) [file pone.0050749.s004.doc]

# **Methods S1.**

### **Preparation of SDECs**

Human endometrial specimens were dissociated mechanically and enzymatically, filtered, isolated by Ficoll gradient, and finally separated into endometrial stromal and glandular epithelial cell fractions as described previously [1]. To remove blood and debris, endometrial tissue samples were rinsed in Dulbecco's Modified Eagle Medium (DMEM) (Sigma-Aldrich, St. Louis, MO, USA) containing a 1% antibiotic-antimycotic supplement (GIBCO, Carlsbad, CA, USA), and 10% FBS (BioWest, Miami, FL, USA) (DMEM+), then weighed and cut into small pieces < one mm^3^. The tissue fragments were digested with 0.2% (wt/vol) collagenase (Wako, Osaka, Japan) and 0.05% DNase I (GIBCO) in DMEM+ for 1.5 h at 37°C on a shaking rotor. Typically, ten mL of the cell digestion medium and one g of the sample were used. After the enzymatic digestion, the tissue digest was filtered through a 40 µm cell strainer (BD Biosciences, Bedford, MA, USA). Most of the stromal cells and blood cells present as single cells or small aggregates passed through the 40 µm cell strainer into a sterile 50 mL polycarbonate tube below, whereas the undigested fragments, mostly made up of glandular clumps, were retained in the strainer. The glandular fragments were recovered from the cell strainer by back-flushing onto ten cm sterile dishes with DMEM+. Stromal cells (prepared in a 50 mL tube) were pelleted by centrifugation for seven min at 440 x g and resuspended in four mL of calcium- and magnesium-free Hanks' Balanced Salt Solutions (HBSS) (Sigma-Aldrich) supplemented with 2% FBS and 10 mM Hepes Buffer Solution (Sigma-Aldrich) (HBSS+). Red blood cells were removed by carefully layering the cell suspension over three mL of Ficoll-Paque PLUS (Amersham Pharmacia Biosciences, Piscataway, NJ, USA) in a 15 mL tube. The solution was centrifuged for 15 min at 780 x g. The medium/Ficoll interface layer, mainly containing stromal and immune cells, was carefully aspirated and washed. Glandular epithelial fragments were collected again and digested with 0.05% trypsin-EDTA solution (Sigma-Aldrich) and 0.05% DNase I by pipetting for five to ten min to dissociate the glands into single cells. Subsequently, the dispersed glandular cells were washed with DMEM+ and filtered through a 40 µm cell strainer for the removal of undigested and sticky glandular tissues to yield a single cell suspension. The mixture of the two fractions was designated as singly dispersed endometrial cells (SDECs). SDECs were subjected to flow cytometry.

### **Isolation and flow cytometric analysis of ESP and EMP cells**

The mixture of two fractions (SDECs) was washed in HBSS+ and suspended at 2 × 10^6^ cells/mL in HBSS+ and stained with 5.0 µg/mL Hoechst33342 (Sigma-Aldrich) for 90 min at 37°C, as described previously[2,3]. To characterize the surface marker of ESP and EMP cells, fluorescein isothiocyanate (FITC)-conjugated or phycoerythrin (PE)-conjugated antibodies for flow cytometry and propidium iodide (PI) (Sigma-Aldrich) were simultaneously added to Hoechst-stained cells suspended in HBSS+. Cells were incubated on ice for 30 min, pelleted, and washed with HBSS+. The antibodies are listed in Table S1. Flow cytometric analyses and cell sorting were performed on a triple laser MoFlo XDP (Beckman Coulter, Nyon, Switzerland) with Summit software (Beckman Coulter). Hoechst 33342 was excited at 350 nm, and the fluorescence emissions were detected using a 450/BP (band pass) 65 and 675/BP30 optical filters for Hoechst blue and Hoechst red, respectively, and a 506 nm long-pass dichroic mirror (Beckman Coulter) to separate the emission wavelengths. Both Hoechst blue and red fluorescence intensities are shown on a linear scale. PI fluorescence was measured through the 630/BP30 after excitation at 488 nm with a sapphire laser, and a live cell gate was defined to exclude PI-positive cells. After collecting 1 × 10^5^ events, the SP and MP populations were defined as previously reported [3]. A new gate was established on this population, as shown in Figure 1A, right upper panel. After the SP and MP populations were defined, these regions were used as live gates to display fluorescence of several cell surface markers of these subsets of cells or to isolate ESP and EMP cells. Samples were analyzed using Kaluza software (Beckman Coulter).

### **Mice**

NOD/SCID/γcnull (NOG) mice were established at the Central Institute of Experimental Animals (Kawasaki, Japan) by backcrossing C57BL/6J-γcnull mice with NOD/Shi-scid mice as reported previously [4]. All mice were provided by the Institute and maintained under specific-pathogen-free conditions in the Animal Center of Keio University School of Medicine in accordance with the guidelines of the facility. The Ethical Review Committee of the Institute approved the experimental protocol.

### **Lentiviral Infection of Transduced ESP and EMP cells and preparation of endometrial grafts**

Freshly isolated ESP and EMP cells were immediately subjected without cell culture to infection with lentivirus by centrifugation at a multiplicity of infection (MOI) of 80, as described previously [5,6]. In brief, 1 × 10^4^ ESP or EMP cells were suspended in 25 µL of DMEM+ in an Eppendorf tube. A tube containing 20 µL of frozen lentivirus stock was quickly thawed in a 37°C water bath and mixed with the ESP or EMP cells; 0.8 mg/mL polybrene (Sigma-Aldrich) was added to the mixture to a final concentration of eight µg/mL. Cells were infected by centrifuging at 1,500 rpm with an Eppendorf centrifuge 5415R (Eppendorf, Hamburg, Germany) for 3 h at room temperature and washed twice with one mL of DMEM+. 1 × 10^4^ infected ESP or EMP cells were mixed with 4.9 × 10^5^ PI-negative SDECs and resuspended in rat-tail collagen (BD Biosciences) neutralized according to the manufacturer's instructions. Cell and collagen suspensions were dispensed in 15 µL aliquots and incubated for 30 min at 37°C as described previously [6]. These mixtures are designated *TdTomato-ESP* and *TdTomato-EMP*, respectively. Mixtures were overlaid with DMEM+ and incubated overnight. The following day, *TdTomato-ESP* or *TdTomato-EMP* was implanted under the kidney capsule of oophorectomized NOG mice. We used two human specimens and the procedure was performed in duplicate or triplicate for each specimen. Thus, a total of five *TdTomato-ESP*s and six *TdTomato-EMP*s were transplanted.

### **Xenotransplantation and hormonal treatment**

We sedated recipient mice with 3% inhaled isoflurane (DS Pharma Animal Health, Osaka, Japan). The right kidney was exteriorized through a dorsal-horizontal incision. A small nick was made on the right flank side of the kidney. The beveled end of PE10 tubing (BD Biosciences) was carefully placed under the capsule, and the tubing was moved around gently to make space as described previously [7]. An endometrial graft was then slowly delivered under the kidney capsule using fine forceps (Dumont #5-45; FST, Vancouver, Canada). The nick was cauterized with low heat. At transplantation, both recipient ovaries were removed to eliminate the influence of endogenous estrogen. The recipient was subcutaneously implanted with two E_2_ pellets (1.5 mg of E_2_ per pellet; Innovative Research of America, Sarasota, FL, USA). A P_4_ pellet (15 mg of P_4_ per pellet; Innovative Research of America) was subcutaneously implanted six weeks after the transplantation. It was anticipated that exposure to sustained levels of E_2_ would provoke proliferative changes in the endometrial transplants, whereas addition of P_4_ would mimic the hormonal environment during the P_4_-dominated secretory phase, inducing secretory changes. These xenotransplanted mice were nephrectomized eight weeks after the transplantation of the endometrial grafts.

### **Histology and immunohistochemistry**

Nephrectomy was performed for morphological and immunohistochemical analyses eight weeks after transplantation. The graft-bearing kidneys excised from NOG mice were embedded in Tissue-Tek OCT compound (Sakura Finetech, Torrance, CA, USA), frozen, and serially sectioned at eight µm using a Leica cryostat (Leica Microsystems, Wetzlar, Germany). We performed histological analysis by staining with H&E (Sigma-Aldrich) for evaluation of the endometrial glandular and stromal structures. Cryosections (eight µm thickness) were transferred to microscope slides, air-dried for 30 min, and washed in PBS twice for three min. Sections were fixed in 4% paraformaldehyde for ten min at room temperature and permeabilized with 0.1% Triton X-100 in PBS for ten min. After blocking with 1% BSA for 30 min, tissue sections were incubated with the pre-titrated primary antibodies listed in Table S2 overnight at 4°C and washed. For co-staining of differentiation markers and TdTomato, rabbit anti-RFP antibody (MBL, Aichi, Japan) was used as the first antibody for TdTomato. The first antibodies were visualized by incubation with secondary antibodies conjugated with goat anti-mouse Alexa Fluor 488 (green) (Life Technologies, Carlsbad, CA, USA) and goat anti-rabbit Alexa Fluor 555 (red) (Cell Signaling Technology, Danvers, MA, USA) for 60 min. After nuclear staining with 1 mg/mL Hoechst 33342 for five min, the slides were washed and mounted with Fluoromount (Diagnostic BioSystems, Pleasanton, CA). The slides were examined by fluorescence microscopy (BIOREVO® BZ-9000, Keyence, Osaka, Japan) and analyzed with BZ-II Image Analysis Application (Keyence). For image cytometry, images were analyzed using TissueQuest software (TissueGnostics, Vienna, Austria). This method has been used previously to quantitate both the numbers of cells and to provide phenotypic characterization of macrophages using CD68 and Iba1 as markers [8,9]. In brief, nuclei were detected by a dissection algorithm in the Hoechst channel. Then, differentiation markers and TdTomato-positive cells were detected by a signal around the nuclei. The signals for the respective primary antibodies were plotted against the nuclear Hoechst signal and against each other to create the scattergrams. We set the cutoff point by referring to the intensity of the mouse kidney parenchyma in each section. Differentiation marker-positive cells were gated and analyzed for TdTomato positivity.

### **Bioluminescence imaging (BLI) and fluorescence imaging (FLI)**

We used a Xenogen-IVIS 100 cooled CCD optical macroscopic imaging system (SC BioScience Corporation, Tokyo, Japan) for BLI and FLI. For *in vivo* BLI, OVX-NOG mice xenotransplanted with lentivirally engineered SDECs were anesthetized with 3% isoflurane and given a retro-orbital injection of D-luciferin (SC BioScience Corporation) (150 mg/kg body weight). The bioluminescence images from the whole bodies of the living mice were acquired after the administration until the maximum intensity was obtained with the field-of-view set at 10 cm, as previously described [1,10]. All images were analyzed with Igor (WaveMetrics, Lake Oswego, OR, USA) and Living Image software (Xenogen, Alameda, CA, USA), and the optical signal intensity was expressed as photon flux, in units of photons/s/cm^2^/steradian. Each image was displayed as a false-color photon-count image superimposed on a grayscale anatomic image. To quantify the measured light, regions of interest (ROI) were defined over the transplanted area and all values were examined from an equal ROI. NOG mouse kidneys excised eight weeks after xenotransplantation were placed on culture dishes and subjected to FLI. Scanning was performed using the DsRed filter (excitation filter 500-550 nm and emission filter 575-650 nm). Non-specific fluorescence was recorded and subtracted from original images by using Living Image software. BLI was also performed on NOG mouse kidneys in the presence of 150 µg/ml D-luciferin as described above.

# **References**

1. Masuda H, Maruyama T, Hiratsu E, Yamane J, Iwanami A, et al. (2007) Noninvasive and real-time assessment of reconstructed functional human endometrium in NOD/SCID/gamma c(null) immunodeficient mice. Proc Natl Acad Sci U S A 104: 1925-1930.

2. Matsuzaki Y, Kinjo K, Mulligan RC, Okano H (2004) Unexpectedly efficient homing capacity of purified murine hematopoietic stem cells. Immunity 20: 87-93.

3. Goodell MA, Brose K, Paradis G, Conner AS, Mulligan RC (1996) Isolation and functional properties of murine hematopoietic stem cells that are replicating in vivo. J Exp Med 183: 1797-1806.

4. Ito M, Hiramatsu H, Kobayashi K, Suzue K, Kawahata M, et al. (2002) NOD/SCID/gamma(c)(null) mouse: an excellent recipient mouse model for engraftment of human cells. Blood 100: 3175-3182.

5. Xin L, Ide H, Kim Y, Dubey P, Witte ON (2003) In vivo regeneration of murine prostate from dissociated cell populations of postnatal epithelia and urogenital sinus mesenchyme. Proc Natl Acad Sci U S A 100 Suppl 1: 11896-11903.

6. Memarzadeh S, Zong Y, Janzen DM, Goldstein AS, Cheng D, et al. (2010) Cell-autonomous activation of the PI3-kinase pathway initiates endometrial cancer from adult uterine epithelium. Proc Natl Acad Sci U S A 107: 17298-17303.

7. Szot GL, Koudria P, Bluestone JA (2007) Transplantation of pancreatic islets into the kidney capsule of diabetic mice. J Vis Exp: 404.

8. Kozakowski N, Bohmig GA, Exner M, Soleiman A, Huttary N, et al. (2009) Monocytes/macrophages in kidney allograft intimal arteritis: no association with markers of humoral rejection or with inferior outcome. Nephrol Dial Transplant 24: 1979-1986.

9. Liesz A, Suri-Payer E, Veltkamp C, Doerr H, Sommer C, et al. (2009) Regulatory T cells are key cerebroprotective immunomodulators in acute experimental stroke. Nat Med 15: 192-199.

10. Okada S, Ishii K, Yamane J, Iwanami A, Ikegami T, et al. (2005) In vivo imaging of engrafted neural stem cells: its application in evaluating the optimal timing of transplantation for spinal cord injury. FASEB J 19: 1839-1841.
